# Impact of Chilling Rate on the Evolution of Volatile and Non-Volatile Compounds in Raw Lamb Meat during Refrigeration

**DOI:** 10.3390/foods10112792

**Published:** 2021-11-12

**Authors:** Can Xiang, Shaobo Li, Huan Liu, Ce Liang, Fei Fang, Dequan Zhang, Zhenyu Wang

**Affiliations:** Key Laboratory of Agro-Products Processing, Institute of Food Science and Technology, Chinese Academy of Agricultural Sciences, Ministry of Agriculture and Rural Affairs, Beijing 100193, China; xiangcan97@126.com (C.X.); lishaobo@caas.cn (S.L.); sd_lh1990@126.com (H.L.); celiang312@163.com (C.L.); fangfei@caas.cn (F.F.); dequan_zhang0118@126.com (D.Z.)

**Keywords:** chilling rate, refrigeration, volatile and non-volatile compound, raw lamb meat

## Abstract

The aim of this study was to investigate the effect of chilling rate (1.44, 22.2, and 32.4 °C/h) on the evolution of volatile and non-volatile compounds in raw lamb meat during refrigeration (1, 24, 72, and 120 h). Through orthogonal projection to latent structure-discriminant analysis, the calculation of odor activity values (OAV > 1) and taste activity values (TAV > 1) analysis, 1-octen-3-ol, (E, E)-2,4-decadienal, nonanal, hexanal, nona-3,5-dien-2-one, 2,3-octanedione, hexanoic acid, 1-nonen-4-ol, aspartate (Asp), Glutamic Acid (Glu), 5′-GMP, 5′-IMP, and 5′-AMP were regarded as differential flavor or taste compounds for raw meat undergone different chilling rates. With a rapid chilling rate at 24 h after slaughter, the contribution of 1-octen-3-ol decreased, but (E, E)-2,4-decadienal increased. Moreover, at 24 h post-mortem, the equivalent umami concentration of Asp, Glu, 5′-GMP, 5′-IMP and 5′-AMP in raw meat were significantly lower at a chilling rate of 1.44 °C/h than 32.4 °C/h (*p* < 0.05). Conclusively, under the rapid chilling rate, more fatty odor and umami compounds accumulated in 24 h aged meat.

## 1. Introduction

Flavor is one of the dominant factors determining the quality of meat and consumer purchasing decisions [1]. Although the flavor of raw meat is slight, it is also an indicator for consumers to evaluate meat freshness and nutrition-related attributes [2]. Meanwhile, some volatile and non-volatile precursors are always produced during refrigeration of raw meat and will determine the cooking quality and sensory attributes of meat products [3]. Therefore, the control and improvement of the volatile and non-volatile compounds in raw meat is a hot topic globally. Many factors influence the volatile and non-volatile compounds in raw meat, such as breeds, gender, age, slaughtering methods, aging time, packaging methods, and storage conditions [4]. Aging after 4 °C chilling is the most popular way to preserve freshness and tenderization of raw meat. Some recent studies reported that compared with traditional 4 °C chilling, fast chilling sped up the reduction of carcass’s temperature and reduced purge losses and cooking losses [5,6]. Rapid chilling of pre-rigor carcasses significantly slowed the post-mortem process [7], reduced the rate of pH decline, and inhibited glycolysis [8,9]. Meanwhile, chilling method affected the degree of lipid oxidation and protein degradation, to produce different volatile and non-volatile compounds such as free fatty acid, oxidative free radical, peptides, and amino acids, which would contribute to cooked meat aroma [1]. Therefore, several researchers raised the possibility that changing the rate of carcass chilling after slaughter may affect meat volatile and non-volatile compounds [10,11]. However, studies only showed that accelerated chilling can improve the physical and chemical properties of meat due to the reduced post-mortem metabolic rate [7,12], the volatile and non-volatile compounds in raw meat cut from the chilled carcass with different chilling rates during refrigeration were not well understood.

The aims of this study were to (1) classify raw meat at different chilling rates and refrigeration times based on analysis of volatile and non-volatile compounds; (2) identify the key and differential volatile and non-volatile compounds in response to different chilling rates, and (3) investigate the evolution of key volatile and non-volatile compounds at different chilling rates during refrigeration.

## 2. Materials and Methods

### 2.1. Sample Preparation

Eighteen 6-month-old Small-Tail sheep (24.27 ± 0.73 kg carcass weight) were randomly obtained from six sheep pens (each pen for three sheep), in Mongolia Grassland Hongbao Food Co., Ltd. (Bayannur, Inner Mongolia, China). All sheep had similar genetic backgrounds and diets. Sheep were slaughtered in accordance with the principles and guidelines established by the Committee for the use of the Institute of Food Science and Technology of the Chinese Academy of Agricultural Sciences. Every three sheep from the same pen were randomly divided into 3 groups (G1, G2, and G3, respectively), resulting in 6 sheep per group. According to the technical specifications for cutting lamb (NY/T 1564–2007) (The Ministry of Agriculture and Rural Affairs of the People’s Republic of China, 2007), similar to backstrap 5101 in the seventh edition of the Australian Meat Manual (Australia, 2005) [13], the left longissimus dorsi was removed and trimmed within 30 min after slaughter. No external fat remained on the longissimus muscle, and the silverskin was removed. Longissimus dorsi muscles were portioned into 4 equally sized segments and allocated to refrigeration of 1, 24, 72, and 120 h from the time of bloodletting, and their initial temperatures were recorded (the thermometer probe was inserted at a depth of about 2 cm). The samples were transferred to chilling rooms within 45 min postmortem, and the thermometer parameters were set to automatically record data every 10 min. G1 samples were chilled at 4 °C, the final temperature was 4 °C, while the rate of chilling was 1.44 °C/h. G2 samples were chilled at −20 °C until the central temperature dropped to −1 °C, and the chilling rate was 22.2 °C/h. G3 samples were chilled at −35 °C, the final temperature was −1 °C, while the rate was 32.4 °C/h. Then the samples were transferred to refrigerate at 4 °C. The samples of the three groups were collected at 1, 24, 72, and 120 h postmortem, dissected into small pieces, snap-frozen in liquid nitrogen, and stored at −80 ℃ until analysis.

### 2.2. Electronic Nose Analysis

The PEN 3.5 (Airsense Analytics GmbH, Schwerin, Germany) system consists of a sampling unit, a sensor chamber containing an array of 10 metal oxide semiconductor type chemical sensors, and pattern recognition software (Winmuster) for data recording and analysis. The Sensors include W1C (sensitive to aromatic components, benzene), W3C (sensitive to aromas, ammonia), W5C (sensitive to short-chain alkane aromatic components), W1S (sensitive to methyl compounds), W2S (sensitive to alcohols, aldehydes, and ketones), W3S (sensitive to long-chain alkanes), W5S (sensitive to nitrogen oxides), W6S (sensitive to hydrides), W1W (sensitive to sulphides) and W2W (sensitive to organic sulphides). PEN 3.5 portable electronic nose was used to obtain the response values of samples [14]. Two grams of minced sample were placed in a 20 mL headspace vial for 30 min at 25 ± 1 °C to generate enough volatile compounds. Samples were tested by headspace sampling, and the sample gas was injected into the sensor chamber at a flow rate of 300 mL/min for 60 s. The response values of each sensor with a detection time of 56–58 s were recorded. The cleaning stage of the electronic nose was conducted to balance sensor signals by indrawing clean air into the sample gas path for 180 s.

### 2.3. Electronic Tongue Analysis

E-tongue (α-Astree; Alpha MOS Company, Toulouse, France) was used to acquire the taste signals of raw meat, including seven liquid cross-sensitive sensors which were AHS (detect sour compounds), ANS (detect sweet compounds), SCS (detect bitter compounds), CTS (detect salty compounds), NMS (detect umami compounds), PKS and CPS (detect compound compounds). Four grams of minced sample were immersed in 20 mL ultra-pure water and homogenized for 1.5 min, then the volume was fixed to 120 mL, ultrasonic extraction for 20 min to extract compounds completely. To protect the sensors, samples need to centrifuge for 15 min (4 °C, 10,000 r/min) and filtered by 0.45 μm membrane, and then 100 mL samples were poured into a 120 mL beaker for detection. The detection time was set to 2 min to ensure that the sensor obtains sufficient taste information for each sample. The sensors were flushed in ultrapure water for 10 s to reach a stable state. The process was repeated seven times for each sample.

### 2.4. Volatile Compounds Analysis

#### 2.4.1. Headspace Solid-Phase Microextraction/Gas Chromatography-Mass Spectrometry (HS-SPME/GC–MS) Analysis

The volatile compounds in raw meat were tested as described by Liu et al. [15] with minor modifications. The volatile compounds were extracted by headspace solid-phase microextraction (HS-SPME). Three grams chopped sample and 1.5 μL 2-methyl-3-heptanone solution (1.60 μg/mL in methanol) as an internal standard were placed in a 20 mL headspace vial. The vials were kept incubated at 50 °C for 20 min. The 65 μm polydimethylsiloxane/divinylbenzene fused silica (PDMS/DVB) coating fiber (Supelco, Inc., Bellefonte, PA, USA) was exposed to the headspace vial for 40 min to absorb the volatile compounds. The fiber was inserted into the sampler of a gas chromatograph (GC) for 2 min for desorption. A gas chromatograph-mass spectrometer (GC-MS) (QP2010plus, Shimadzu) equipped with a DB-WAX (30 m × 0.25 mm × 0.25 μm) was used to detect volatile compounds in raw meat. Helium has a flow rate of 1 mL/min as a carrier. The temperatures of the injector and ion source were 200 °C, and 250 °C, respectively. The initial temperature of the oven was 40 °C, and the heating procedure of the oven was held at 40 °C for 3 min, ramped up to 120 °C at 5 °C/min, ramped up to 200 °C at 10 °C/min, and held for 13 min. Mass spectrometry was performed in electron collision mode (EI) with a voltage of 70 eV and a full scan mode of 35–500 *m*/*z*.

According to the computer spectrum library (NIST11, NIST11s), compounds with the matching degree of volatile compounds greater than 80 were identified by MS, and the highest matching degree was 100. LRIs were analyzed according to the retention times of external standard n-alkanes (C_7_-C_40_) under the same GC-MS-detection conditions, and LRIs were compared with n-alkanes (C_7_-C_40_) reported in the literature to identify the aromatic compounds [16]. Semi-quantitative analysis of volatile compounds in raw meat was carried out using 2-methyl-3-heptanone as an internal standard. The concentration of volatile compounds was calculated according to the peak area ratio and the concentration of 2-methyl-3-heptanone.

#### 2.4.2. Calculations of Odor Activity Values (OAVs)

To evaluate the contribution of volatile compounds to raw meat, odor activity value (OAV) and contribution rate were calculated. The ratio of concentration to perceptual threshold presented OAV [17], the ratio of the OAV to the total OAVs represented the contribution rate of these compounds. Compounds with OAV greater than 1 may be the main contributors, while compounds with OAV less than 1 may be the secondary contributors.

### 2.5. Free Amino Acids Analysis

The contents of free amino acids (FAAs) of raw meat were measured as described by Zhang et al. [15,18] with some modifications. The FAAs contents in the samples were detected by using L8900 Amino Acid Analyzer (Hitachi High-Technologies Corporation, Tokyo, Japan). Two grams of minced meat were added with 15 mL HCl (0.1 mol/L). The mixture was homogenized for 2 min and centrifuged (8500 r/min, 4 °C) for 15 min. One milliliter of supernatant was added to 1 mL of sulfosalicylic acid (8%) and centrifuged again (10,000 r/min, 15 min, 4 °C). Then, 1 mL of supernatant was dried under nitrogen (40 °C) and dissolved in 1 mL of hydrochloric acid solution (0.02 mol/L). The filtration solution was tested after filtration through a pinhole filtration membrane (0.22 μm).

### 2.6. Nucleotide Analysis

The contents of nucleotides in raw meat were detected by using high-performance liquid chromatography (HPLC) equipped with a TSK gel ODS-80TM (4.6 mm × 250 mm × 5 μm) (Tosoh quartz Co., Ltd., Tainan, Japan) [15,19]. Ten grams of minced meat were added with 30 mL HClO_4_ (5%). The mixture was homogenized (10,000 r/min, 2 × 20 s) and centrifuged (8500 r/min, 4 °C) for 10 min, and the supernatant was filtered through a medium speed filter paper. After that, the pH of the filtrate was adjusted to 5.4, and the volume was adjusted to 100 mL. The filtrates were tested after filtration through a pinhole filtration membrane (0.22 μm).

The column and UV wavelengths of HPLC were 30 °C and 254 nm, respectively. The injection volume was 10 μL, and the rate of flow was 0.6 mL/min. The mobile phase solvents were methanol (A) and 0.05 mol/L potassium dihydrogen phosphate buffer solution (pH = 5.4) (B). The gradient elution was carried out with binary mobile solvents, in which the ratio of methanol to 0.05 mol/L potassium dihydrogen phosphate was as follows: 0–10 min: 0%/100%, 11–17 min: 10%/90%, 18–25 min: 0%/100%, the detection lasted for 25 min. 

### 2.7. Calculations of Equivalent Umami Concentrations (EUCs) and Taste Active Values (TAVs)

The EUCs of taste compounds were calculated as follows:

Y = Σa_i_b_i_ + 1218(Σa_i_b_i_)Σa_j_b_j_ (1)

Y was the EUC of the raw meat (g MSG/100 g). ai and aj were the concentrations of umami amino acids (Glu or Asp) (g/100 g) and taste nucleotides (5′-AMP, 5′-IMP, 5′-GMP) (g/100 g), respectively. bi and bj were the relative umami coefficients of the umami amino acids to MSG (Glu: 1; Asp: 0.077) and the relative umami coefficients of taste nucleotides to IMP (5′-AMP: 0.18; 5′IMP: 1; 5′-GMP: 2.3), respectively.

The TAVs of the taste compounds in raw meat were calculated as the ratio of the concentration of each compound to the taste threshold. A TAV greater than 1 indicated that the compound made a significant contribution to the taste of the sample. Conversely, it had a secondary impact on the taste.

### 2.8. Statistical Analysis

All statistical analyses of volatile and non-volatile compounds of raw meat were conducted using SPSS 26.0 software (IBM Corporation, Armonk, NY, USA). Data were compared between groups using a general linear model analysis of variance. Duncan’s multiple range test was applied to analyze differences (*p* < 0.05). Orthogonal projection to latent structure-discriminant analysis (OPLS-DA) was fulfilled by SIMCA 14.1 (MKS Data Analytics Solutions, Umea, Sweden). Results were expressed as the means ± standard deviation (*n* = 6).

## 3. Results and Discussion

### 3.1. Volatile and Non-Volatile Compounds Identification of Raw Meat with Different Chilling Rates and Different Refrigeration Times

#### 3.1.1. Effect of Chilaling Rates

The OPLS-DA model was used as an internal prediction validation method in which Q^2^ represented the degree of prediction of the model, R^2^X on the *X*-axis represented the model’s goodness of fit (GOF), and R^2^Y on the *Y*-axis represented the GOF of the model.

**Figure 1:**
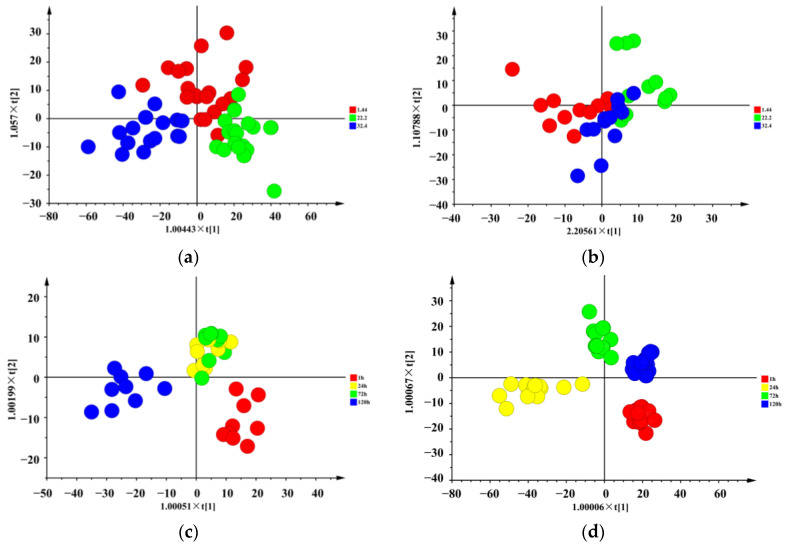
OPLS-DA score plots of volatile compounds and non-volatile compounds in raw meat at different chilling rates and different refrigeration times. (**a**) OPLS-DA score plot of volatile compounds at different chilling rates. (**b**) OPLS-DA score plot of non-volatile compounds at different chilling rates. (**c**) OPLS-DA score plot of volatile compounds at different refrigeration times. (**d**) OPLS-DA score plot of non-volatile compounds at different refrigeration times.

The OPLS-DA results for the classification of volatile and non-volatile compounds in raw meat at different chilling rates were shown in Figure 1. For the identification of volatile compounds at different chilling rates (Figure 1a), the values of R^2^X, R^2^Y, and Q^2^ were 0.846, 0.65, and 0.543, respectively. Correspondingly, for the non-volatile compounds at different chilling rates (Figure 1b), the values of R^2^X, R^2^Y, and Q^2^ were 0.981, 0.264, and 0.211, respectively. The non-volatile compounds of samples with chilling rates of 1.44, 22.2, and 32.4 °C/h partially clustered together, indicating similarities within the samples that made separation difficult, while for the volatile compounds of samples with chilling rates of 1.44, 22.2, and 32.4 °C/h were separated completely. Regarding the volatile compounds, in the scoring plots of samples with chilling rates of 1.44, 22.2, and 32.4 °C/h, good inter-class variation could be observed along the first predicted component (Figure 1a). Meanwhile, negative scores correspond to samples with a chilling rate of 32.4 °C/h and positive scores of 22.2 °C/h. Samples with a chilling rate of 1.44 °C/h had a positive distribution on t[2]. Correspondingly, the score plot for non-volatile compounds (Figure 1b) showed a grouping of samples with a chilling rate of 32.4 °C/h distributed prevalently as negative scores on t[2], while the best separation between 1.44 and 22.2 °C/h samples was provided by t[1].

The result showed that increasing the chilling rate altered the volatile and non-volatile compounds of raw meat, especially the volatile compounds. Previous studies reported that a rapid chilling rate could reduce the rate of pH decline [20], which affected the glycolysis and enzymatic activities [21,22,23]. Furtherly, the rapid chilling could affect the concentration of volatile and non-volatile precursors [24,25], resulting in different flavors of raw meat at different chilling rates. Also, protein oxidation and lipid oxidation have a great influence on the flavor and consumer acceptance of raw meat [26]. Storing meat at lower temperatures slowed down the oxidation and spoilage process [27,28], which remained and even improved raw meat flavor.

#### 3.1.2. Effect of Refrigeration Times

For the classification of volatile compounds in raw meat with different refrigeration times (Figure 1c), the values of R^2^X, R^2^Y, and Q^2^ were 0.969, 0.924, and 0.852, respectively. Correspondingly, for non-volatile compounds in raw meat with different refrigeration times (Figure 1d), the values of R^2^X, R^2^Y, and Q^2^ were 0.917, 0.851, and 0.605, respectively. Four distinct clusters were also identified along the two first predictive components, t[1] and t[2], of the score plot for volatile compounds modeled according to refrigeration times (Figure 1c). 24, 72, and 120 h subjects were well discriminated along the t[1]. Variability between 1 and 120 h subjects was collected by t[2]. In the score plot of the non-volatile compounds calibration model (Figure 1d), excellent inter-class comparability between the 1 and 120 h samples could be observed for the first predictive component, where negative scores correspond to 120 h samples and positive scores to 1 h samples. Score plot for the non-volatile compounds also showed a grouping of 24 h and 72 h samples that distributed prevalently as positive scores on the t[2].

The transformation of muscle into meat after slaughter is accompanied by many biochemical and structural activities. These processes have been shown to be influenced by post-slaughter handling [29,30] and to affect the volatile and non-volatile compounds, tenderness, color, and other qualities of the raw meat [31]. The conduction of volatile and non-volatile compounds at different times after slaughter were affected by protein oxidation and lipid oxidation [32]. The formation of post-mortem peptides, free fatty acids, and oxidative free radicals led to the generation of flavor precursors, which in turn produced different volatile and non-volatile compounds [1,33].

### 3.2. Identification of Key Volatile and Non-Volatile Compounds in Raw Meat during Refrigeration

#### 3.2.1. Key Volatile and Non-Volatile Compounds

**Figure 2:**
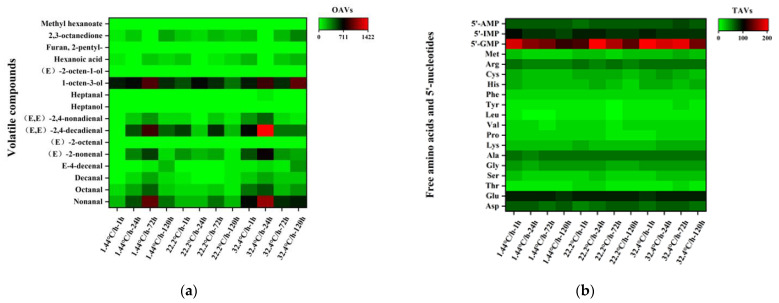
OAVs and TAVs of volatile compounds and non-volatile compounds in raw meat at different chilling rates during refrigeration. (**a**) OAVs of volatile compounds. (**b**) TAVs of non-volatile compounds.

**Figure 3:**
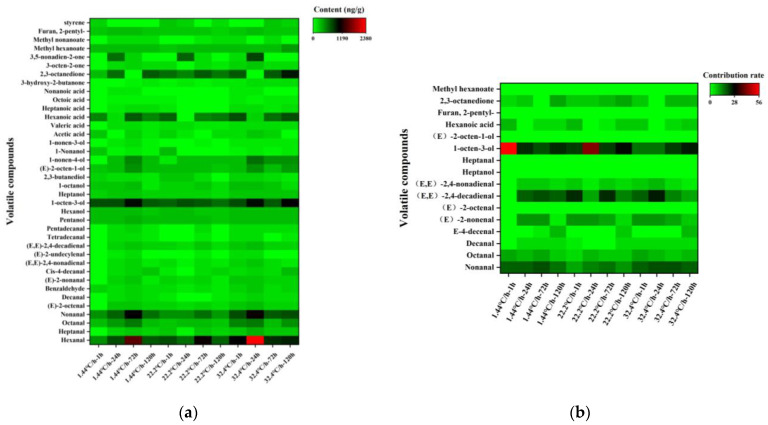
Contents and Contribution rates of volatile compounds in raw meat at different chilling rates during refrigeration. (**a**) Contents of volatile compounds. (**b**) Contribution rates of volatile compounds.

Volatile compounds with odor-activity values (OAVs) > 1 made a dominant contribution to raw meat, while compounds with OAVs < 1 contributed less [16]. The concentration of a volatile compound was divided by the published odor threshold in water to determine its OAV. As shown in Figure 2a, 16 volatile compounds with OAVs > 1 were set out below: nonanal, octanal, decanal, (E)-4-decenal, (E)-2-nonanal, (E)-2-octenal, (E, E)-2,4-decadienal, (E, E)-2,4-nonadienal, heptanol, heptanal, 1-octen-3-ol, (E)-2-octen-1-ol, hexanoic acid, 2-pentylfuran, 2,3-octanedione, and methyl hexanoate. To intuitively explain the contribution of volatile compounds to raw meat, the contribution rate was used to elaborate. As shown in Figure 3b, the higher contribution rates of volatile compounds in raw meat were 1-octen-3-ol (19.31–56.21%), (E, E)-2,4-decadienal (10.7–25.34%), and nonanal (9.50–20.34%), and they accounted for almost 80% of the contribution rates. 1-octen-3-ol, (E, E)-2,4-decadienal, and nonanal could be recognized as key volatile compounds in raw meat during refrigeration. Consistently, several studies demonstrated that nonanal, (E, E)-2,4-decadienal, and 1-octen-3-ol had higher OAVs in raw and cooked meat [15,34,35]. Alcohols were typical volatile markers of raw and unprocessed meat. They were mainly oxidative decomposition products of fat, especially 1-octen-3-ol, which had a mushroom odor and was reported to be generated by β-oxidation [36,37,38]. The aldehydes with the largest contribution rate of volatile compounds were (E, E)-2, 4-decadienal, and nonanal, which had the odor of fat [17,39].

Taste activity values (TAVs) identified the main non-volatile compounds. As shown in Figure 2b, aspartic acid (Asp), Glu, 5′-GMP, 5′-IMP, and 5′-AMP had a TAV greater than 1. Therefore, they were key non-volatile compounds in raw meat. Glu, 5′-GMP and 5′-IMP were widely regarded as monosodium glutamate (MSG)-like components that gave a pleasant broth or a rich meaty odor [39,40]. Previous studies showed that nucleotides, such as 5′-GMP, 5′-IMP, and 5′-AMP, usually be added to raw meat in order to improve the sweetness and umami taste, while some free amino acids such as Asp and Glu were also umami-taste-related free amino acids [41,42,43]. In addition, nucleotides, and umami amino acids (UAA) created synergies to enhance the taste of meat. In agreement with previous studies [44], MSG-like components were the key non-volatile components in raw meat.

#### 3.2.2. Differential Metabolites in Raw Meat: Effect of Chilling Rates

**Figure 4:**
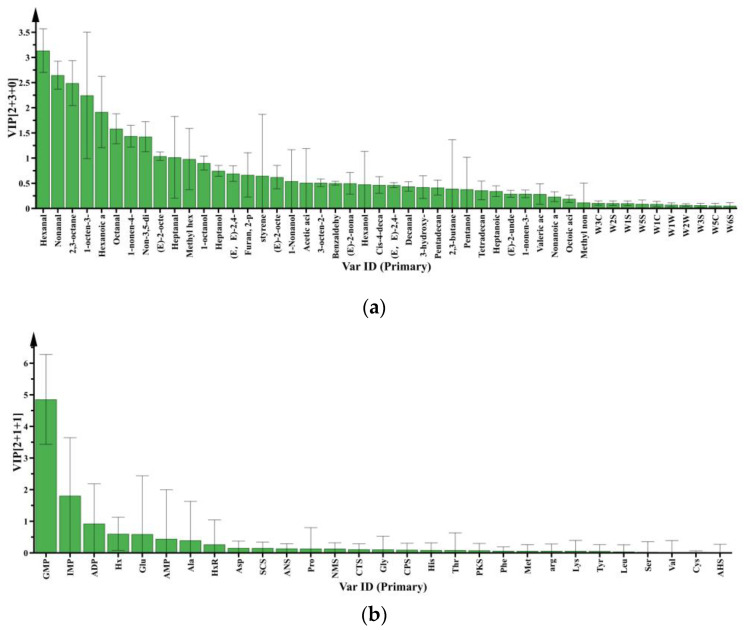

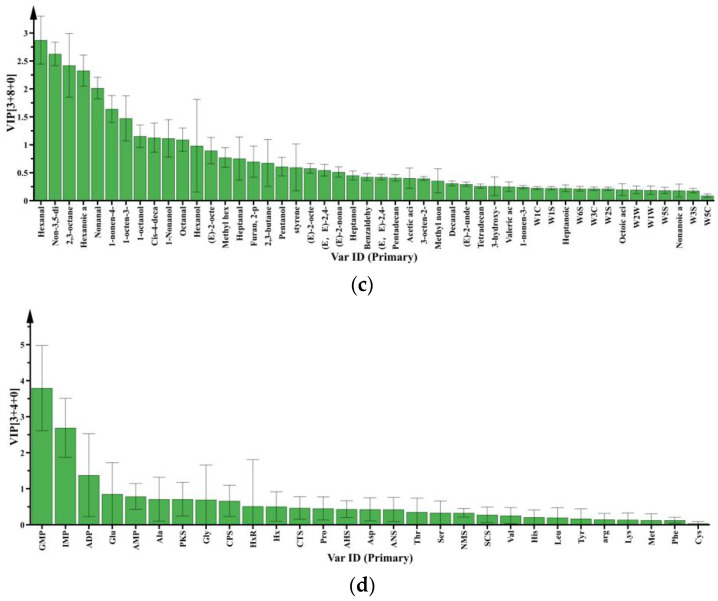
VIP values of volatile compounds and non-volatile compounds in raw meat at different chilling rates and different refrigeration times. (**a**) VIP values of volatile compounds at different chilling rates. (**b**) VIP values of non-volatile compounds at different chilling rates. (**c**) VIP values of volatile compounds at different refrigeration times. (**d**) VIP values of non-volatile compounds at different refrigeration times.

Six variables were regarded as critical volatile compounds in raw meat at different chilling rates, as their variable importance in projection (VIP) were all above 1.5 (Figure 4a). The name and VIP values of these six compounds were: hexanal (3.14), 2,3-octanedione (2.49), nonanal (2.65), 1-octen-3-ol (2.24), hexanoic acid (1.92), octanal (1.58). According to Figure 4b, among the non-volatile compounds, 5’-inosinic acid (IMP) and 5’-guanylic acid (GMP) were differential compounds at different chilling rates, and the VIP values were 4.85 and 1.81, respectively. Therefore, hexanal, 2,3-octanedione, nonanal, 1-octen-3-ol, hexanoic acid, octanal, 5′-IMP, and 5′-GMP were the critical volatile metabolites in raw meat at different chilling rates.

The rapid chilling could significantly retard the postmortem process [7] and decrease the rate of pH decline [8]. In addition, lower ambient temperatures decreased the lipid oxidation [45], especially for raw meat stored at −30 and −80 °C [27]. Hexanal, 2,3-octanedione, nonanal, 1-octen-3-ol, hexanoic acid, and octanal were all regarded as the products of lipid oxidation [46], so they might be the differential volatile compounds in raw meat under different chilling rates. For non-volatile compounds, the formation of IMP was controlled by an essential enzyme adenylate deaminase (AMPD) in adenosine triphosphate (ATP) degradation [47]. The hydrolysis of IMP was controlled by acid phosphatases (ACP) and alkaline phosphatases (ALP). Lower refrigeration temperatures inhibited the activity of glycolytic enzymes and reduced the rate of ATP turnover, resulting in different IMP and GMP contents at different chilling rates [48].

#### 3.2.3. Differential Metabolites in Raw Meat: Effect of Refrigeration Time

During refrigeration, volatile compounds with VIP values greater than 1.5 in raw meat included hexanal (2.87), nona-3,5-dien-2-one (2.63), 2,3-octanedione (2.42), hexanoic acid (2.32), nonanal (2.01), and 1-nonen-4-ol (1.64). For non-volatile compounds, both GMP (3.79) and IMP (2.69) were deemed to be significant with predictive VIP > 1.5 (Figure 4c,d). Metabolites including hexanal, nona-3,5-dien-2-one, 2,3-octanedione, hexanoic acid, nonanal, 1-nonen-4-ol, 5′-GMP, and 5′-IMP were detected as the differential non-volatile compounds at different refrigeration times. Moreover, 2,3-octanedione, nonanal, hexanoic acid, and 1-octen-3-ol have been reported as volatile compounds affected by aging in raw meat associated with lipid oxidation [49]. IMP was the differential non-volatile compounds in raw meat at different refrigeration times, and decreased with the increase of inosine, hypoxanthine, and ribose [50].

### 3.3. Changes in Key Volatile and Non-Volatile Compounds in Raw Meat at Different Chilling Rates and Different Refrigeration Times

#### 3.3.1. Changes in Key Volatile Compounds

As shown in Table 1 and Figure 3a, 38 volatile compounds were detected in raw meat, mainly including aldehydes, alcohols, acids, esters, ketones, heterocyclic, and alkanes. Hexanal (526.32–2375.77 ng/g) had the most abundant content, followed by 1-octen-3-ol (398.33–916.86 ng/g), 2,3-octanedione (127.39–858.26 ng/g), and nonanal (192.54–1095.56 ng/g). Based on OAVs and the contribution rates, 1-octen-3-ol, (E, E)-2,4-decadienal, and nonanal were recognized as key volatile compounds in raw meat during refrigeration with different chilling rates. In terms of contribution rate, the contribution rate of 1-octene-3-ol at 72 h post-slaughter was significantly lower (*p* < 0.05) than that at other post-slaughter times at chilling rates of 1.44 °C/h, but the contribution rate of aldehydes was significantly higher (*p* < 0.05). When the chilling rate was 32.4 °C/h, the contribution rate of aldehydes at 24 h post-mortem was significantly higher (*p* < 0.05) than that at other post-mortem hours, while 1-octen-3-ol was significantly lower (*p* < 0.05). (E, E)-2,4-decadienal was the highest contributing aldehyde, which enhanced the aroma of raw meat [51]. The results also showed (E, E)-2, 4-decadienal was the predominant volatile compound at 72 h post-slaughter when the chilling rate was 1.44 °C/h and 22.2 °C/h, while (E, E)-2, 4-decadienal was the largest contributor of the volatile compound at 24 h when the rate was 32.4 °C/h. This might be due to the delayed phase of the decrease in ATP concentration associated with a rapid chilling rate (32.4 °C/h). ATP remained almost constant during chilling at −20 °C or lower [52], at which temperature the breakdown of ribonucleotides, protein hydrolysis, and glycolysis would be inhibited. Conversely, some oil-derived volatiles, including (E, E)-2, 4-decadienal, were increased through the breakdown of ribonucleotides, protein hydrolysis, and glycolysis [53,54,55]. Therefore, the contribution rate of (E, E)-2,4-decadienal in raw meat at 24 h post-slaughter was higher at 32.4 °C/h than 1.44 °C/h.

The contents of 1-octen-3-ol, (E, E)-2,4-decadienal, and nonanal in raw meat first increased and then decreased during refrigeration, and their OAVs had the same trend. Similar results were obtained from studies on Jingyuan meat [35]. 1-octen-3-ol was mainly derived from linoleic acid when amino acids and ribose interact during lipid oxidation [56], and aldehydes also could be formed through lipid oxidation [57]. Therefore, the contents of 1-octen-3-ol, (E, E)-2,4-decadienal and nonanal increased in the early post-slaughter period, which might be due to lipid oxidation of raw meat. Generally, the fatty flavor was richer under the rapid chilling rate, especially at 24 h postmortem. In addition, more aromatic compounds were observed in lamb meat at 72 h postmortem with a chilling rate of 1.44 °C/h and at 24 h with a chilling rate of 32.4 °C/h.

#### 3.3.2. Changes in Key Non-Volatile Compounds

As shown in Table 2, the main free amino acids in raw meat were Glu (24.85–67.72 mg/100 g), Ala (22.67–41.55 mg/100 g), which were umami and sweet, respectively. The content of 5′-nucleotides and the corresponding nucleotides of raw meat at three chilling rates during refrigeration were shown in Table 3. 5′-GMP was the most abundant nucleotide in raw meat, ranging from 1797.17 to 2555.08 mg/100 g. The 5′-IMP content was ranged from 153.41 to 722.99 mg/100 g, and 5′-ADP content was ranged from 64.72 to 146.69 mg/100 g. AMP, Hx, and HxR were ranged from 16.78 to 47.79 mg/100 g, 16.71 to 33.35 mg/100 g, and 12.72 to 20.01 mg/100 g, respectively. Based on TAVs, Asp, Glu, 5′-GMP, 5′-IMP, and 5′-AMP were the key non-volatile compounds in raw meat during refrigeration with different chilling rates.

**Figure 5:**
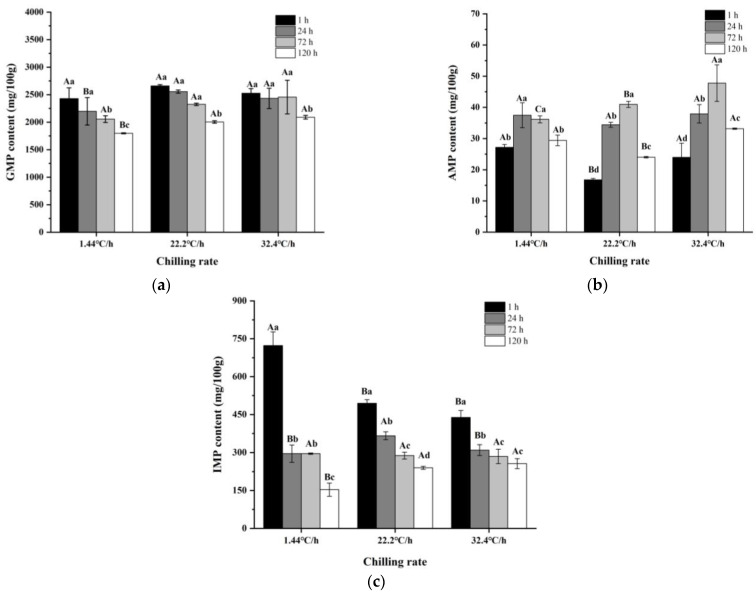
Contents of 5′-nucleotide in raw meat at different chilling rates during refrigeration. (**a**) Contents of 5′-guanylic acid (5’-GMP). (**b**) Contents of 5′-Adenosine monophosphate (5′-AMP). (**c**) Contents of 5′-inosine monophosphate (5’-IMP).

**Figure 6:**
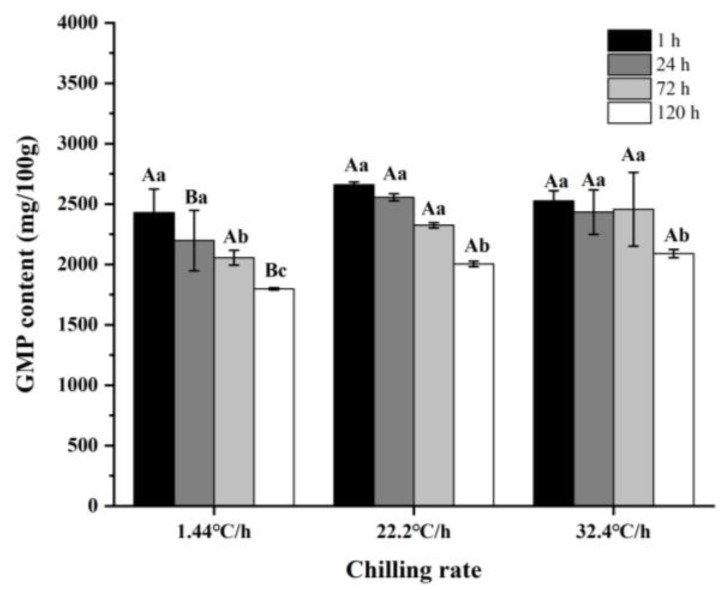
EUCs of non-volatile compounds in raw meat at different chilling rates during refrigeration.

As shown in Figure 5, the rate of decrease in IMP and GMP contents with a chilling rate of 32.4 °C/h was significantly lower (*p* < 0.05) than that with 1.44 °C/h. When the chilling rate was 1.44 °C/h, the contents of UAAs at 24 h after slaughter were significantly lower (*p* < 0.05) than that at other times. However, at 22.2 and 32.4 °C/h, the contents of UAAs were the lowest (*p* < 0.05) at 72 h after slaughter. To get a better view of the umami intensity of raw meat, the EUCs of umami amino acids and taste nucleotides were calculated. As could be clearly observed in Figure 6, the EUCs and contents of UAAs showed the same trend at different chilling rates. The reduction in the contents of UAAs and FAAs might be related to the enhanced glycolysis and the rapid decrease in pH and ATP content during refrigeration. Several studies showed that rapid chilling rate could significantly inhibit pH decline, ATP consumption and suppress glycolysis [21,58,59].

As refrigeration time was extended, the contents of 5′-IMP and 5′-GMP decreased. The content of 5′-AMP increased first and then decreased, while both the contents of UAAs and FAAs and the EUCs of MSG-like components first decreased and then increased during refrigeration. During the conversion of animal muscle into meat, protein hydrolysis was released by the action of muscle proteases (calpain, histopeptidase, and multicatalytic protease complexes), which would produce intermediate polypeptides. Under the role of exopeptidases (dipeptidyl peptidase and tripeptidyl peptidase), aminopeptidase, and carboxypeptidase, the peptide was further hydrolyzed to produce small peptides and free amino acids [60,61]. UAAs and FAAs contents had a similar trend of decreasing and then increasing during rigor mortis, the reduction in the contents might be attributed to the enhanced glycolysis and the rapid decrease in pH and ATP content after slaughter [62]. The activation of enzymes such as calpain accelerated the hydrolysis of proteins, leading to an increase in the contents of free amino acids. ATP in raw meat was decomposed into adenosine diphosphate (ADP) under the action of ATPase, and then ADP was catabolized to AMP and IMP by the action of creatine kinase and adenylate dehydrogenase. IMP was not stable and prone to produce Inosine (HxR) and hypoxanthine (Hx) by nucleoside hydrolase [50], which caused the IMP to continue to break down during refrigeration.

## 4. Conclusions

In summary, both chilling rates and refrigeration times affected the volatile and non-volatile compounds of raw meat. The present study also potentially established a method to identify raw meat with different chilling rates and refrigeration times based on volatile and non-volatile compounds analysis combined with OPLS-DA. 1-octen-3-ol, (E, E)-2,4-decadienal, Asp, Glu, 5′-GMP, 5′-IMP, and 5′-AMP were identified as the key volatile and non-volatile compounds. The rapid chilling rate resulted in more aromatic compounds in raw meat, especially for samples refrigerated for 24 h. Raw lamb meat chilled at 1.44 °C/h had more aromatic and umami compounds at 72 h after slaughter, and those chilled at 32.4 °C/h had more aromatic and umami compounds at 24 h. It was concluded that rapid chilling could be a potential method to preserve and even enhance the flavor of raw meat. This result might be further explained by the ability of rapid chilling to retard adenosine triphosphate hydrolysis, decelerate protein derogation, lipolysis, and fat oxidation.

## Figures and Tables

**Table 1:** Identification of volatile compounds in raw meat.

Compound ^a^	LRI ^b^		Identification ^e^	Compound ^a^	LRI ^b^		Identification ^e^
	Calculated ^c^	Literature ^d^			Calculated ^c^	Literature ^d^	
Hexanal	1076	1080	MS, LRI, S	2,3-Butanediol	1571	1570	MS, LRI
Heptanal	1168	1170	MS, LRI	2-Octen-1-ol, (E)-	1606	1603	MS, LRI, S
Octanal	1269	1275	MS, LRI, S	1-Nonen-4-ol	1635	- ^f^	MS
Nonanal	1376	1380	MS, LRI	1-Nonanol	1657	1656	MS, LRI, S
(E)-2-Octenal	1412	1416	MS, LRI, S	1-Nonen-3-ol	1747	1555	MS, LRI
Decanal	1481	1483	MS, LRI, S	acetic acid	1442	1441	MS, LRI
Benzaldehyde	1502	1495	MS, LRI, S	Pentanoic acid	1729	1734	MS, LRI
(E)-2-Nonenal	1518	1514	MS, LRI, S	Hexanoic acid	1835	1834	MS, LRI, S
cis-4-Decenal	1525	1542	MS, LRI	Heptanoic acid	1966	1967	MS, LRI
(E, E)-2,4-Nonadienal	1682	1687	MS, LRI, S	Octanoic acid	2068	2072	MS, LRI
2-Undecenal	1739	1755	MS, LRI	Nonanoic acid	2171	2174	MS, LRI
(E, E)-2,4-Decadienal	1752	1763	MS, LRI, S	3-hydroxy-2-butanone	1265	1259	MS, LRI
Tetradecanal	1900	1908	MS, LRI	2,3-Octanedione	1311	1325	MS, LRI
Pentadecanal	2037	2042	MS, LRI	3-Octen-2-one	1393	1414	MS, LRI
1-Pentanol	1262	1261	MS, LRI	3,5-Nonadien-2-one	1801	- ^f^	MS
1-Hexanol	1354	1356	MS, LRI, S	Hexanoic acid, methyl ester	1170	1177	MS, LRI
1-Octen-3-ol	1447	1447	MS, LRI, S	Nonanoic acid, methyl ester	1477	1481	MS, LRI
1-Heptanol	1452	1458	MS, LRI, S	Furan, 2-pentyl-	1202	1213	MS, LRI, S
1-Octanol	1553	1554	MS, LRI, S	styrene	1234	1242	MS, LRI

^a^ Volatile compound detected in raw meat. ^b^ Linear retention index. ^c^ Calculated data based on n-alkanes (C_7_-C_40_). ^d^ Reported data. ^e^ MS, mass spectrum; LRI, linear retention index; S, confirmed by authentic aroma standards. ^f^ Not identified.

**Table 2:** Contents (mg/100 g) of free amino acid in raw meat at different chilling rates during refrigeration.

Content (mg/100 g)	Flavor Trait	Chilling Rate	Storage Time				*p*-Value		
			1 h	24 h	72 h	120 h	Storage Time	Chilling Rate	Storage Time × Chilling Rate
**Asp**	Umami	1.44 °C/h	4.6 ± 0.74 ^Aa^	3.52 ± 1.23 ^Aa^	3.36 ± 0.35 ^ABa^	2.77 ± 0.09 ^Ba^	0.004	0.004	0.000
		22.2 °C/h	3.21 ± 0.17 ^Ab^	1.1 ± 0.17 ^Cc^	3.46 ± 0.28 ^Aa^	2.85 ± 1.00 ^Ba^			
		32.4 °C/h	1.63 ± 0.14 ^Ac^	2.2 ± 0.54 ^Ab^	1.37 ± 0.10 ^Bb^	1.78 ± 0.17 ^Ab^			
**Glu**	Umami	1.44 °C/h	40.67 ± 4.75 ^Bb^	33.81 ± 1.55 ^Cc^	57.08 ± 6.69 ^Aa^	62.76 ± 10.62 ^Aa^	0.000	0.000	0.000
		22.2 °C/h	66.2 ± 4.44 ^Aa^	67.72 ± 4.05 ^Aa^	41.78 ± 3.23 ^Bb^	67.17 ± 3.48 ^Aa^			
		32.4 °C/h	48.66 ± 0.22 ^Ab^	55.38 ± 8.30 ^Ab^	24.85 ± 6.05 ^Bc^	44.21 ± 7.59 ^Ab^			
**Thr**	Sweet	1.44 °C/h	2.83 ± 0.36 ^ABa^	2.27 ± 0.37 ^Bb^	1.85 ± 0.02 ^Cb^	3.29 ± 0.64 ^Ab^	0.000	0.001	0.017
		22.2 °C/h	2.69 ± 0.24 ^Ca^	3.55 ± 0.32 ^Ba^	2.94 ± 0.12 ^Ca^	4.19 ± 0.16 ^Aa^			
		32.4 °C/h	2.47 ± 0.07 ^Ba^	3.49 ± 0.72 ^Aa^	1.81 ± 0.35 ^Cb^	3.03 ± 0.48 ^Ab^			
**Gly**	Sweet	1.44 °C/h	14.68 ± 0.97 ^Aa^	11.8 ± 1.48 ^Bc^	9.38 ± 0.12 ^Bb^	12.83 ± 2.09 ^ABb^	0.010	0.000	0.002
		22.2 °C/h	10.47 ± 0.66 ^Bb^	14.68 ± 0.86 ^Ab^	8.45 ± 0.49 ^Cb^	14.63 ± 0.54 ^Aa^			
		32.4 °C/h	14.33 ± 0.23 ^Ba^	18.35 ± 2.66 ^Aa^	17.38 ± 2.95 ^Aa^	14.94 ± 2.19 ^ABa^			
**Ala**	Sweet	1.44 °C/h	41.55 ± 3.66 ^Aa^	34.14 ± 3.52 ^Bb^	30.81 ± 0.13 ^Ca^	34.43 ± 5.69 ^Ba^	0.086	0.001	0.014
		22.2 °C/h	22.67 ± 1.23 ^Bc^	30.07 ± 2.12 ^Ac^	27.61 ± 1.46 ^Ab^	30.65 ± 1.93 ^Ab^			
		32.4 °C/h	27.84 ± 0.42 ^Bb^	38.54 ± 6.05 ^Aa^	29.14 ± 4.28 ^ABab^	31.12 ± 5.13 ^ABab^			
**Lys**	Sweet/Bitter	1.44 °C/h	2.29 ± 0.29 ^Aa^	1.72± 0.19 ^Bb^	1.91 ± 0.02 ^Bb^	2.98 ± 0.53 ^Ab^	0.000	0.004	0.012
		22.2 °C/h	2.11 ± 0.15 ^Ca^	2.82 ± 0.11 ^Ba^	2.45 ± 0.41 ^Ba^	3.54 ± 0.04 ^Aa^			
		32.4 °C/h	1.84 ± 0.19 ^Bb^	2.53 ± 0.42 ^Aa^	2.48 ± 0.23 ^Aa^	2.73 ± 0.45 ^Ab^			
**Pro**	Sweet/Bitter	1.44 °C/h	4.4 ± 0.06 ^Aa^	4.33 ± 0.22 ^Ab^	3.93 ± 0.15 ^Ba^	4.94 ± 0.86 ^Ab^	0.000	0.000	0.002
		22.2 °C/h	4.51 ± 0.14 ^Ba^	6.04 ± 0.54 ^Aa^	3.61 ± 0.49 ^Bab^	6.7 ± 0.55 ^Aa^			
		32.4 °C/h	4.17 ± 0.23 ^Bb^	5.4 ± 0.81 ^Aab^	3.22 ± 0.1 ^Cb^	4.33 ± 0.27 ^Bb^			
**Val**	Sweet/Bitter	1.44 °C/h	3.03 ± 0.42 ^Aa^	2.53 ± 0.32 ^Bb^	1.33 ± 0.21 ^Cb^	2.81 ± 0.47 ^Bb^	0.000	0.000	0.003
		22.2 °C/h	2.44 ± 0.13 ^Cb^	3.08 ± 0.2 ^Ba^	2.35 ± 0.06 ^Ca^	3.43 ± 0.2 ^Aa^			
		32.4 °C/h	1.86 ± 0.14 ^Bc^	2.29 ± 0.41 ^Ab^	2.12 ± 0.31 ^Aa^	2.38 ± 0.4 ^Ab^			
**Leu**	Bitter	1.44 °C/h	3.82 ± 0.4 ^Aa^	3.15 ± 0.37 ^Aa^	1.82 ± 0.15 ^Bc^	3.73 ± 0.64 ^Ab^	0.000	0.231	0.000
		22.2 °C/h	2.51 ± 0.22 ^Bb^	3.35 ± 0.25 ^Aa^	2.91 ± 0.12 ^Bb^	4.95 ± 0.34 ^Aa^			
		32.4 °C/h	2.28 ± 0.15 ^Bb^	3.18 ± 0.48 ^Aa^	3.6 ± 0.48 ^Aa^	3.6 ± 0.66 ^Ab^			
**Tyr**	Bitter	1.44 °C/h	2.62 ± 0.24 ^Aa^	2.22 ± 0.1 ^Bb^	1.66 ± 0.04 ^Cb^	2.85 ± 0.45 ^Ab^	0.000	0.004	0.002
		22.2 °C/h	2.09 ± 0.14 ^Cb^	2.91 ± 0.19 ^Ba^	2.7 ± 0.16 ^Ba^	3.82 ± 0.19 ^Aa^			
		32.4 °C/h	2.05 ± 0.3 ^Bb^	2.97 ± 0.36 ^Aa^	3.08 ± 0.58 ^Aa^	3.06 ± 0.52 ^Ab^			
**Phe**	Bitter	1.44 °C/h	2.48 ± 0.33 ^Ba^	2.68 ± 0.06 ^Ba^	2.57 ± 0.34 ^Bb^	3.27 ± 0.34 ^Aab^	0.000	0.183	0.034
		22.2 °C/h	2.25 ± 0.17 ^Ca^	2.88 ± 0.16 ^Ba^	2.87 ± 0.25 ^Bab^	3.72 ± 0.29 ^Aa^			
		32.4 °C/h	2.39 ± 0.12 ^Ba^	2.96 ± 0.13 ^Aa^	3.18 ± 0.12 ^Aa^	3.14 ± 0.34 ^Ab^			
**His**	Bitter	1.44 °C/h	1.74 ± 0.25 ^Aa^	0.88 ± 0.32 ^Bb^	0.53 ± 0.21 ^Bb^	1.41 ± 0.51 ^ABa^	0.015	0.005	0.006
		22.2 °C/h	0.63 ± 0.39 ^Bb^	1.02 ± 0.22 ^Aab^	0.33 ± 0.03 ^Cb^	0.93 ± 0.05 ^Ab^			
		32.4 °C/h	0.74 ± 0.49 ^Bb^	1.37 ± 0.14 ^Aa^	1.17 ± 0.17 ^Aa^	1.24 ± 0.23 ^Aa^			
**Arg**	Bitter/Sweet	1.44 °C/h	5.02 ± 0.56 ^Aa^	3.97 ± 0.47 ^Cb^	4.51 ± 0.13 ^Ba^	5.29 ± 0.91 ^Aab^	0.009	0.162	0.008
		22.2 °C/h	3.53 ± 0.24 ^Bb^	4.91 ± 0.36 ^Aa^	3.38 ± 0.25 ^Bb^	5.45 ± 0.32 ^Aa^			
		32.4 °C/h	3.86 ± 0.86 ^Ab^	4.22 ± 0.71 ^Aab^	4.52 ± 0.49 ^Aa^	4.37 ± 0.58 ^Ab^			
**Cys**	Bitter/Sweet/Sulfurous	1.44 °C/h	0.13 ± 0.04 ^Aa^	0.08 ± 0.03 ^Ac^	0.19 ± 0 ^Aa^	0.23 ± 0.07 ^Aa^	0.043	0.061	0.001
		22.2 °C/h	0.06 ± 0.05 ^Ac^	0.13 ± 0.03 ^Ab^	0.07 ± 0.08 ^Ab^	0.18 ± 0.07 ^Ab^			
		32.4 °C/h	0.09 ± 0 ^Db^	0.19 ± 0.03 ^Aa^	0.12 ± 0 ^Bb^	0.07 ± 0 ^Cc^			
**Met**	Bitter/Sweet/Sulfurous	1.44 °C/h	0.95 ± 0.06 ^Aa^	0.79 ± 0.16 ^Ab^	0.45 ± 0.05 ^Bc^	1.12 ± 0.18 ^Ab^	0.000	0.000	0.000
		22.2 °C/h	0.65 ± 0.21 ^Cb^	1.11 ± 0.11 ^Ba^	0.9 ± 0.04 ^Db^	2.29 ± 0.18 ^Aa^			
		32.4 °C/h	0.79 ± 0.09 ^Bb^	1.2 ± 0.17 ^Aa^	1.28 ± 0.12 ^Aa^	1.46 ± 0.29 ^Ab^			
**FAA**	UN	1.44 °C/h	135.18 ± 13.53 ^Aa^	111.48 ± 6.93 ^Bb^	123.98 ± 5.93 ^Aa^	148.89 ± 24.62 ^Ab^	0.001	0.445	0.016
		22.2 °C/h	128.77 ± 7.69 ^Bb^	149.35 ± 9.17 ^Aa^	108.93 ± 6.03 ^Cb^	159.9 ± 7.23 ^Aa^			
		32.4 °C/h	118.59 ± 0.75 ^Bc^	148.82 ± 22.3 ^Aa^	102.98 ± 15.4 ^Cb^	125.65 ± 19.67 ^Ac^			

^a, b, c^ Different lowercase letters indicate significant differences between chilling rates (*p* < 0.05). ^A, B, C, D^ Different capital letters indicate significant differences between refrigeration times (*p* < 0.05).

**Table 3:** Contents (mg/100 g) of 5′-nucleotides in raw meat at different chilling rates during refrigeration.

Content (mg/100 g)	Flavor Trait	Chilling Rate	Storage Time				*p*-Value		
			1 h	24 h	72 h	120 h	Storage Time	Chilling Rate	Storage Time × Chilling Rate
**ADP**	UN	1.44 °C/h	64.72 ± 6.13 ^Cb^	103.76 ± 7.11 ^Ba^	113.22 ± 6.96 ^Ba^	140.56 ± 0.89 ^Aa^	0.000	0.048	0.002
		22.2 °C/h	106.13 ± 2.11 ^Ba^	71.98 ± 0.83 ^Cb^	85.12 ± 0.98 ^Cb^	146.69 ± 14.59 ^Aa^			
		32.4 °C/h	106.76 ± 12.69 ^Ba^	76.95 ± 8.73 ^Cb^	102.29 ± 11.1 ^Ba^	140.09 ± 11.13 ^Aa^			
**AMP**	Umami	1.44 °C/h	27.19 ± 0.9 ^Ca^	37.48 ± 3.98 ^Aa^	36.17 ± 1.12 ^Ac^	29.41 ± 1.69 ^Bb^	0.000	0.000	0.000
		22.2 °C/h	16.78 ± 0.41 ^Dc^	34.42 ± 0.82 ^Ba^	40.97 ± 0.97 ^Ab^	24 ± 0.24 ^Cc^			
		32.4 °C/h	23.99 ± 4.49 ^Db^	37.94 ± 2.95 ^Ba^	47.79 ± 5.87 ^Aa^	33.16 ± 0.21 ^Ca^			
**GMP**	Umami/Sweet	1.44 °C/h	2430.04 ± 194.1 ^Ac^	2197.66 ± 249.65 ^Ac^	2056.68 ± 61.18 ^Bb^	1797.17 ± 8.92 ^Cb^	0.000	0.000	0.000
		22.2 °C/h	2660.13 ± 22.5 ^Aa^	2555.08 ± 29.53 ^Ba^	2324.4 ± 22.27 ^Ca^	2003.88 ± 22.97 ^Da^			
		32.4 °C/h	2526.15 ± 83.82 ^Ab^	2432.85 ± 185.17 ^Ab^	2457.26 ± 305.49 ^Aa^	2090.01 ± 33.49 ^Ba^			
**IMP**	Umami	1.44 °C/h	722.99 ± 54.07 ^Aa^	296 ± 34.4 ^Bb^	295.93 ± 2.99 ^Ba^	153.41 ± 26.25 ^Cb^	0.000	0.000	0.000
		22.2 °C/h	494.55 ± 14.17 ^Ab^	366.11 ± 15.58 ^Ba^	288.36 ± 13.61 ^Ca^	239.94 ± 5.81 ^Da^			
		32.4 °C/h	438.62 ± 27.43 ^Ab^	309.9 ± 21.54 ^Ba^	284.74 ± 28.52 ^Ca^	256.37± 19.75 ^Da^			
**Hx**	Bitter	1.44 °C/h	23.61 ± 1.05 ^Aa^	25.87 ± 5.42 ^Aa^	23.98 ± 2.34 ^Ac^	24.94 ± 0.21 ^Ab^	0.000	0.000	0.054
		22.2 °C/h	22.21 ± 6.07 ^Ba^	31.95 ± 1.3 ^Aa^	33.35 ± 0.75 ^Aa^	23.39 ± 0.22 ^Bb^			
		32.4 °C/h	25.97 ± 1.34 ^Aa^	28.26 ± 2.06 ^Aa^	27.2 ± 2.87 ^Ab^	28.65 ± 7.99 ^Aa^			

^a, b, c^ Different lowercase letters indicate significant differences between chilling rates (*p* < 0.05). ^A, B, C, D^ Different capital letters indicate significant differences between refrigeration times (*p* < 0.05). ND: not detected.

## Data Availability

The data presented in this study are available on request from the corresponding author.
